# Comparisons of Audiologic Characteristics in Patients with Continuous and Intermittent Tinnitus

**DOI:** 10.3390/clinpract14040111

**Published:** 2024-07-11

**Authors:** Seok Hwan Chung, Sung Soo Kim, Sang Hoon Kim, Seung Geun Yeo

**Affiliations:** 1 Department of Otorhinolaryngology, Head and Neck Surgery, College of Medicine, Kyung Hee University Hospital, Seoul 02447, Republic of Korea; seokhwanchung@naver.com (S.H.C.); hoon0700@naver.com (S.H.K.); 2 Department of Biochemistry and Molecular Biology, School of Medicine, Kyung Hee University, Seoul 02447, Republic of Korea; sgskim@khu.ac.kr

**Keywords:** continuous tinnitus, intermittent tinnitus, pure-tone audiometry, auditory brainstem response, otoacoustic emissions

## Abstract

Background: No studies to date have compared audiologic characteristics in patients with continuous and intermittent tinnitus. The present study classified tinnitus patients into continuous and intermittent groups based on tinnitus duration and compared their audiologic characteristics. Methods: This study enrolled 604 patients with tinnitus from January 2019 to December 2022. Clinical manifestations, PTA results, the frequency and loudness of tinnitus, ABR, DPOAE, and TEOAE tests were compared in patients with continuous and intermittent tinnitus. Results: Of the 604 patients, 231 (38.2%) had continuous and 373 (61.8%) had intermittent tinnitus. There were no significant between-group differences in otologic symptoms, tinnitus onomatopoeia. PTA showed that hearing thresholds, except at 125 Hz, were significantly higher in patients with continuous rather than intermittent tinnitus. The loudness of tinnitus was significantly greater in patients with continuous rather than intermittent tinnitus. ABR tests showed that the absolute latency of wave V was significantly longer in continuous than in intermittent tinnitus. Signal-to-noise ratios on TEOAE tests were significantly lower in patients with continuous rather than intermittent tinnitus at all frequencies tested (1, 1.5, 2, 3, and 4 kHz). Response rates to sound stimuli at all frequencies, except for 1 kHz, were significantly lower on DPOAE tests in patients with continuous rather than intermittent tinnitus. Conclusions: Continuous tinnitus is more common in males, more persistent over time, and is associated with a higher rate of hearing loss. In contrast, intermittent tinnitus is more common in women, appears acutely, and is associated with a relatively lower rate of hearing loss. Based on the findings of the current paper, it seems that audiologic characteristics may differ between patients with continuous and intermittent tinnitus.

## 1. Introduction

Tinnitus is a subjective symptom of hearing in the inner ear or brain, independent of external sounds. Tinnitus may be accompanied by hearing loss, ear fullness, and/or vertigo, and severe tinnitus can lead to neuropsychiatric conditions such as depression, anxiety, and insomnia, significantly impacting quality of life [1,2,3]. The prevalence of tinnitus is around 11.6% to 17.0% in men, 10.5% to 16.2% in women, and 12.6% to 16.5% in the general population [4]. In Korea, the prevalence of tinnitus is 19.7%, with 29.3% of tinnitus patients aged ≥12 years experiencing daily discomfort [5]. The prevalence of tinnitus is gradually increasing due to aging of the population and increased exposure to noise [6,7].

Tinnitus can manifest in various forms, including conductive tinnitus, sensorineural hearing-loss-related tinnitus, and vascular tinnitus. Conductive tinnitus arises from middle-ear factors like ear infections, issues with the eardrum and ossicular chain, and glomus tumors. Sensorineural tinnitus is linked with sensorineural hearing loss, which is the most prevalent tinnitus type. This type can be connected to conditions like presbycusis, metabolic disorders such as diabetes mellitus, hypothyroidism, dyslipidemia, anemia, deficiencies in vitamins and minerals, and exposure to loud noises. Vascular tinnitus is triggered by turbulence in blood flow that impacts the cochlea [8,9]. 

Although studies have evaluated the pathogenesis, risk factors, and treatment of tinnitus, few studies to date have compared the classifications and characteristics of continuous and intermittent tinnitus. About 42% of patients diagnosed with tinnitus have continuous tinnitus, whereas about 25% have intermittent tinnitus. The prevalence of continuous tinnitus is higher in subjects exposed to noise, suggesting that continuous tinnitus is associated with hearing loss [10]. In contrast, intermittent tinnitus is more prevalent than continuous tinnitus among individuals with normal hearing and tinnitus (68% vs. 32%) [11]. In another study, the ratio of intermittent tinnitus was higher than that of continuous tinnitus [12]. Moreover, continuous tinnitus is associated with a longer duration and causes greater discomfort than intermittent tinnitus [13,14].

Based on the authors investigations, no studies to date have examined the audiologic characteristics of continuous and intermittent tinnitus. The present study was designed to classify tinnitus patients into those with continuous tinnitus and those with intermittent tinnitus, based on the duration of tinnitus, and to compare the auditory characteristics of these two groups.

## 2. Materials and Methods

### 2.1. Study Design

The study population consisted of 1478 patients with tinnitus who visited the Otolaryngology Outpatient Department at our hospital and underwent audiologic examination from January 2019 to December 2022. Patients with incomplete medical records regarding the characteristics of tinnitus and those with missing test results were excluded. In addition, patients who had a perforated tympanic membrane, had ear trauma in the past, and were taking ototoxic drugs were excluded from the study.

The medical records of included patients were reviewed retrospectively. Factors recorded included patient age, gender, sidedness of tinnitus, type of tinnitus sound, persistence of tinnitus, duration from onset to evaluation, baseline factors, and accompanying otologic symptoms. Tinnitus was classified as either continuous or intermittent based on its duration. Continuous tinnitus was defined as tinnitus that persisted in constant size and pattern regardless of time and place. On the other hand, intermittent tinnitus was defined as tinnitus that varied in size and pattern depending on time and place. Tinnitus was divided into acute tinnitus and chronic tinnitus according to the onset period. Acute tinnitus was defined as lasting <3 months and chronic tinnitus was defined as lasting >3 months [8]. Underlying diseases included hypertension, diabetes, and hyperlipidemia, whereas accompanying otologic symptoms included hearing loss, hyperacusis, ear fullness, dizziness, and autophonia. All patients were evaluated by pure-tone audiometry (PTA), the frequency and loudness of tinnitus, auditory brainstem response (ABR), distortion produced otoacoustic emission (DPOAE), and transient evoked otoacoustic emission (TEOAE) tests. 

The study protocol was approved by the Institutional Review Board of our hospital, which waived the requirement for informed consent due to the retrospective design of this study (IRB No 2019-07-065).

### 2.2. Pure-Tone Audiometry (PTA)

The hearing threshold on PTA (Otometrics Madsen Astera 2, Natus, Taastrup, Denmark) was determined using the 6-division method, which involved averaging the thresholds at 250 Hz, 500 Hz, 1000 Hz, 2000 Hz, 4000 Hz, and 8000 Hz. In patients who reported bilateral tinnitus on questionnaires and those with bilateral tinnitus confirmed by tinnitograms, the sidedness of tinnitus was based on the higher hearing threshold observed on PTA.

### 2.3. Impedance Audiometry (IA)

Impedance audiometry (Otometrics Madsen Zodiac 901, Natus, Taastrup, Denmark) was performed in all patients, and if the result showed type B or C, it was excluded from our study.

### 2.4. Frequency and Loudness of Tinnitus

We asked the patient to respond when they perceived the presented sound to resemble their tinnitus. At the frequency the patient identified as similar to their tinnitus, we introduced noise at an intensity 10 dBHL below their threshold. We raised the intensity in 2 dBHL increments until the patients felt that the intensity matched their perceived tinnitus [11,15]. 

### 2.5. Auditory Brainstem Responses (ABRs), Distortion Product Otoacoustic Emissions (DPOAEs), and Transient Evoked Otoacoustic Emissions (TEOAEs)

ABR (Navigator Pro, Natus, Taastrup, Denmark) was performed to assess damage to the auditory nerve and the auditory conduction pathway in the brainstem. In our hospital, electrodes were placed on the forehead (active), mastoid or earlobe of the test ear (reference), and another mastoid (ground). The stimulus intensity started at 70–90 dB nHL and was adjusted to find the threshold. Polarity settings used alternating clicks to minimize artifacts.

Waves I and II have been shown to originate from the distal and proximal portions, respectively, of the auditory nerve, with waves III, IV, and V originating from the cochlear nucleus, superior olivary complex, and lateral lemniscus, respectively [16,17]. The evaluation included six scales: I latency, III latency, V latency, I-III IPL (interpeak latency), III-V IPL, and I-V IPL. DPOAE and TEOAE tests (DP Echoport, Otodynamics, Hatfield, England) were performed to evaluate the function of the cochlear outer hair cells [18,19]. DPOAE measured the response of outer hair cells to stimuli at frequencies of 1, 2, 3, 4, and 6 kHz, with responses characterized as negative (0) or positive (1). For 1, 1.5, 2, 3, and 4 kHz on TEOAE, we established Signal-to-Noise Ratios (SNRs), and any SNR greater than 3 dB was considered abnormal [19,20].

### 2.6. Statistical Analysis

Categorical variables were compared using chi-square tests, whereas continuous variables were compared using Student’s *t*-tests. All statistical analyses were performed using SPSS V22.0 (IBM Corp., Armonk, NY, USA) software, with *p*-values < 0.05 considered statistically significant.

## 3. Results

A total of 1478 patients with tinnitus visited the Otolaryngology Outpatient Department at our hospital and underwent audiologic examination from January 2019 to December 2022. The records of 489 of these patients were incomplete, with the remaining 989 patients classified as having continuous (n = 414) or intermittent (n = 575) tinnitus. Of these groups, 183 and 202 patients, respectively, met the exclusion criteria and were excluded. The remaining 604 patients included 231 with continuous and 373 with intermittent tinnitus. Analysis by gender showed that intermittent tinnitus was significantly more prevalent in women, whereas continuous tinnitus was significantly more prevalent in men (*p* < 0.05). Age was not significantly associated with the persistence of tinnitus, with no distinction between groups aged <60 and ≥60 years (*p* > 0.05). The laterality of tinnitus did not differ significantly between the groups of patients with continuous and intermittent tinnitus (*p* > 0.05). Chronic occurrences were more frequent than acute occurrences in patients with continuous tinnitus, whereas acute occurrences were more common than chronic occurrences in patients with intermittent tinnitus (*p* < 0.05). Previous medical history, the presence of symptoms accompanying tinnitus, and the persistence of tinnitus did not differ significantly between patients with continuous and intermittent tinnitus (*p* > 0.05). Further analysis of the accompanying symptoms showed that in patients with hearing loss, continuous tinnitus was significantly more frequent than intermittent tinnitus (*p* < 0.05). None of the other otologic symptoms, however, differed significantly between patients with continuous and intermittent tinnitus (*p* > 0.05) (Table 1).

PTA found that hearing thresholds, except at 125 Hz, were significantly higher in patients with continuous rather than intermittent tinnitus (*p* < 0.05). Additionally, the average hearing threshold was significantly higher for continuous rather than for intermittent tinnitus (*p* < 0.05) (Table 2). There were no significant differences in frequency between continuous and intermittent tinnitus (*p* > 0.05), whereas the loudness of tinnitus was significantly greater in patients with continuous rather than intermittent tinnitus (*p* < 0.0001) (Table 3).

ABR tests found that the absolute latency of wave V was significantly longer in patients with continuous rather than intermittent tinnitus (*p* < 0.05). The absolute latencies of waves I and III were also longer in patients with continuous tinnitus, but these differences were not statistically significant (*p* > 0.05). Evaluation showed that I-V IPLs were significantly longer in patients with continuous rather than intermittent tinnitus (*p* < 0.05). In addition, the I-III and III-V IPLs were greater in patients with continuous rather than intermittent tinnitus, but these differences did not differ significantly (*p* > 0.05) (Table 4).

TEOAE tests found that the Signal-to-Noise Ratio at all frequencies tested (1, 1.5, 2, 3, and 4 kHz) was significantly lower in patients with continuous rather than intermittent tinnitus (*p* < 0.05) (Table 5). DPOAE tests showed that the rate of response to the sound stimulus at all stimulating frequencies, except for 1 kHz, was significantly lower in patients with continuous rather than intermittent tinnitus (*p* < 0.05) (Table 5).

## 4. Discussion

The causes and clinical manifestations of tinnitus vary widely, making accurate evaluation and diagnosis challenging. Because hearing loss has been reported to be a common underlying cause of tinnitus [6,19,20], appropriate audiologic evaluation plays a crucial role in the diagnosis of tinnitus [21,22]. The present study, therefore, compared and analyzed the audiological characteristics of patients with continuous and intermittent tinnitus using PTA, the frequency and loudness of tinnitus, ABR, DPOAE, and TEOAE tests.

Previous studies comparing the demographic characteristics of patients with continuous and intermittent tinnitus have reported that continuous tinnitus is more prevalent in men, whereas intermittent tinnitus is more prevalent in women [15]. Similar findings were observed in the present study. Additionally, the present study found that continuous tinnitus tended to manifest as a chronic condition, whereas intermittent tinnitus was more likely to occur acutely. The present study also found that the onset duration was significantly shorter in patients with intermittent rather than continuous tinnitus, in agreement with previous findings [15].

In the present study, patients with continuous tinnitus and those with intermittent tinnitus were found to have mild hearing loss. Furthermore, hearing thresholds at all frequencies were significantly higher in patients with continuous rather than intermittent tinnitus, indicating a close relationship between the persistence of tinnitus and hearing loss. That is, a longer duration of tinnitus was associated with more severe hearing loss. The frequency of tinnitus reported by patients was found to be closely associated with the frequency range of decreased auditory functions in the cochlea [23,24,25,26,27]. The present study found that the frequency of continuous tinnitus was higher than that of intermittent tinnitus, although the difference was not statistically significant. These results suggest that patients with continuous tinnitus have a greater decrease in auditory function at higher frequency ranges than patients with intermittent tinnitus, but the differences were not statistically significant. In addition, higher rates of tinnitus have been found to correlate with a higher prevalence of hearing loss [28,29]. Consistent with these findings, the present study found that the frequency of continuous tinnitus was higher than that of intermittent tinnitus, accompanied by a higher incidence of hearing loss on PTA. The perceived loudness of tinnitus is closely related to the subjective discomfort experienced by patients, with increased loudness having a greater impact on patient quality of life, including increased rates of negative outcomes, such as depression and insomnia [30,31]. The present study found that loudness was significantly greater in patients with continuous rather than intermittent tinnitus, suggesting that continuous tinnitus is more frequently accompanied by the discomfort of tinnitus and the potential for depression.

ABR is a test method that evaluates the integrity of the auditory pathway, including the auditory nerve and central auditory conduction. Previous studies have found a statistically significant delay in wave V has been observed in patients with continuous tinnitus, perhaps due to a relatively higher degree of damage occurring in the midbrain regions involved in tinnitus regulation, leading to a lack of balance [32]. Other previous studies have also suggested a relationship between the duration of tinnitus and delayed wave V on ABR, as significant delays in wave V were observed in patients with continuous tinnitus [33]. The present results also showed a statistically significant delay in wave V of ABR testing in patients with continuous tinnitus, consistent with previous findings. Taken together, these findings suggest that abnormalities in the auditory pathway, including the cochlea and beyond, are more frequent in patients with continuous than with intermittent tinnitus. Furthermore, increased damage to the central nervous system involved in tinnitus regulation is associated with a longer duration of tinnitus.

DPOAE is more specific in assessing the function of the high-frequency region responsible for hearing [34]. Previous studies have shown that response rates in otoacoustic emission testing are significantly lower in patients with rather than without tinnitus, even if they had normal hearing [35,36]. Several other studies have also reported lower results on otoacoustic emission testing in patients with tinnitus than in normal controls [37,38,39,40], suggesting that tinnitus is closely associated with damage to the outer hair cells of the cochlea. The present study found that response levels in TEOAE tests were significantly lower at all frequencies in patients with continuous rather than intermittent tinnitus, and that the response levels in DPOAE tests were significantly lower at 2, 3, 4, and 6 kHz in patients with continuous tinnitus. These results suggested that damage to the outer hair cells of the cochlea is more severe in patients with continuous than with intermittent tinnitus and that damage to the outer hair cells is a factor influencing the duration of tinnitus.

Although this study confirmed audiologic characteristics depending on tinnitus persistence, it did not compare the subjective discomfort of patients with continuous tinnitus versus intermittent tinnitus. We believe that it would be better to conduct further research using questionnaires like the Tinnitus Handicap Inventory (THI). Another limitation of this study is that, unlike the results of our study, some recent studies have found that there are no differences in the results of OAE and ABR when comparing groups with tinnitus and groups without tinnitus [41,42]. Although the total number of patients is smaller compared to our study, we believe that additional research is needed as this is contradictory to our study. 

## 5. Conclusions

This study showed the occurrence of audiological differences between patients with continuous tinnitus and those with intermittent tinnitus. Continuous tinnitus is more common in males, more persistent over time, and associated with a higher rate of hearing loss. In contrast, intermittent tinnitus is more common in women, appears acutely, and is associated with a relatively lower rate of hearing loss. PTA showed that hearing thresholds at all frequencies are elevated in patients with continuous tinnitus. ABR testing showed delays in all waves in patients with continuous tinnitus, as well as specific delays in V waves and I-V IPLs. Additionally, OAE showed that the rate of response to stimulating sound is lower in patients with continuous rather than intermittent tinnitus.

## Figures and Tables

**Table 1:** Demographic characteristics of patients with continuous and intermittent tinnitus.

Characteristics	Continuous (n = 231)	Intermittent (n = 373)	*p*-Value
Age (years)	Age ≤ 60:Age > 60 = 136:95	Age ≤ 60:Age > 60 = 226:147	0.6759
Gender	M:F = 106:125	M:F = 103:270	* <0.0001
Laterality	Rt:Lt = 100:131	Rt:Lt = 163:210	0.9214
Duration of tinnitus	Acute:Chronic = 80:151	Acute:Chronic = 187:186	* <0.0001
Hypertension	21.65% (50/231)	24.4% (71/373)	0.5275
Diabetes mellitus	4.76% (11/231)	3.49% (13/373)	0.4196
Dyslipidemia	3.46% (8/231)	9% (2.41/373)	0.9254
No underlying disease	70.03% (162/231)	69.70% (260/373)	0.3295
Hearing disturbance	41.99% (97/231)	31.18% (116/373)	* 0.0069
Hyperacusis	0% (0/231)	0.27% (1/373)	0.4303
Ear fullness	31.6% (73/231)	31.99% (119/373)	0.9209
Vertigo	20.35% (47/231)	19.09% (71/373)	0.7045
Autophonia	7.79% (18/231)	12.63% (47/373)	0.0623

* *p* < 0.05.

**Table 2:** Mean and standard deviation of pure-tone audiometry results in patients with continuous and intermittent tinnitus.

Frequency	Continuous (n = 231)	Intermittent (n = 373)	*p*-Value
125 Hz	21.41 ± 13.42	20.91 ± 13.75	0.3792
250 Hz	21.28 ± 14.84	19.18 ± 14.12	* 0.0349
500 Hz	21.86 ± 15.79	18.53 ± 13.79	* 0.0072
1000 Hz	25.28 ± 16.54	20.95 ± 15.07	* 0.0004
2000 Hz	27.84 ± 17.31	22.33 ± 16.00	* <0.0001
3000 Hz	31.39 ± 18.26	23.87 ± 23.87	* <0.0001
4000 Hz	39.81 ± 19.04	30.09 ± 20.60	* <0.0001
8000 Hz	50.41 ± 22.36	39.46 ± 25.53	* <0.0001
Average	27.98 ± 15.21	22.53 ± 14.50	* <0.0001

Hz; Hertz; * *p* < 0.05.

**Table 3:** Mean and standard deviation of frequency and loudness of tinnitus among patients with continuous and intermittent tinnitus.

	Continuous (n = 231)	Intermittent (n = 373)	*p*-Value
Frequency	5764.10 ± 3001.15	5203.13 ± 3460.42	0.1467
Loudness	52.21 ± 21.07	41.13 ± 21.26	* <0.0001

* *p* < 0.05.

**Table 4:** Mean and standard deviation ABR results in patients with continuous and intermittent tinnitus.

	Continuous (n = 231)	Intermittent (n = 373)	*p*-Value
I latency	1.64 ± 0.20	1.62 ± 0.19	0.2331
III latency	3.84 ± 0.23	3.80 ± 0.21	0.0620
V latency	5.75 ± 0.27	5.69 ± 0.26	* 0.0083
I–III IPL	2.19 ± 0.15	2.18 ± 0.14	0.3579
III–V IPL	1.91 ± 0.16	1.91 ± 0.27	0.0745
I–V IPL	4.11 ± 0.21	4.07 ± 0.20	* 0.0234

* *p* < 0.05.

**Table 5:** TEOAE results showing Signal-to-Noise Ratios and DPOAE results showing response rates in patients with continuous and intermittent tinnitus.

TEOAE				DPOAE			
kHz	Continuous (n = 231)	Intermittent (n = 373)	*p*-Value	kHz	Continuous (n = 231)	Intermittent (n = 373)	*p*-Value
1	3.38 ± 9.97	5.22 ± 9.70	* 0.0242	1	58.33	62.74	0.1827
1.5	8.54 ± 10.32	11.00 ± 10.13	* 0.0007	2	62.1	72.05	* 0.0016
2	7.24 ± 9.71	10.30 ± 9.59	* <0.0001	3	43.01	57.22	* <0.0001
3	3.56 ± 8.87	6.82 ± 9.28	* <0.0001	4	36.02	53.23	* <0.0001
4	0.93 ± 8.22	4.52 ± 9.24	* <0.0001	6	29.03	44.11	* <0.0001

* *p* < 0.05. TEOAE; transient evoked otoacoustic emission, DPOAE; distortion product otoacoustic emission, kHz; kiloHertz.

## Data Availability

The raw data supporting the conclusions of this article will be made available by the authors on request.
